# Clozapine – The Neglected Gold Standard: A Case Demonstrating Significant Recovery After Late Initiation in Treatment-Resistant Schizophrenia

**DOI:** 10.1192/j.eurpsy.2026.12177

**Published:** 2026-07-31

**Authors:** M. Derganc, A. Pirtovšek Šavs

**Affiliations:** Univerzitetna psihiatrična Klinika Ljubljana, Ljubljana, Slovenia

## Abstract

**Introduction:**

Clozapine remains the most effective medication for the treatment of treatment-resistant schizophrenia, as confirmed by numerous studies and extensive clinical experience. Nevertheless, in clinical practice it is often introduced too late or not at all. The main reasons for this include fear of adverse effects, lack of clinical experience, and the more demanding process of initiation and monitoring. Among the most significant potential complications are neutropenia, delayed intestinal motility, myocarditis, sedation, tachycardia, and orthostatic hypotension. However, when properly monitored, clozapine treatment can profoundly enhance quality of life, even in patients who have failed to respond to multiple prior antipsychotic therapies.

**Objectives:**

-

**Methods:**

-

**Results:**

The patient was first hospitalized at the age of 28 due to a psychotic episode. She was treated with risperidone, but residual delusions persisted, and a diagnosis of paranoid schizophrenia was confirmed. After discharge, due to cognitive decline, she was unable to continue her studies. She lived with her parents and took part in rehabilitation programs and community activities organized by a Slovenian non-governmental organization for mental health.

At the age of 30, she was hospitalized again due to severe distress related to feelings of aging. Over the following years, she was treated with several antipsychotics, including quetiapine, paliperidone, and amisulpride, but without significant clinical improvement. She spent most of her time at home, rarely leaving the apartment. Her only regular social contact was a volunteer who occasionally accompanied her on walks.

She lived in constant fear, interpreting global events such as the COVID-19 pandemic and the wars in Ukraine and Gaza as being personally connected to her. At the age of 50, she was hospitalized once more, and clozapine treatment was initiated. After six months of therapy, her condition improved markedly. She achieved a satisfactory remission and described an inner sense of calm. She began taking daily walks again and became more engaged in everyday life. Her family described her as calm, stable, and more emotionally present.

**Conclusions:**

Clozapine remains a cornerstone in the treatment of resistant schizophrenia, yet it is still underused due to persistent prejudices and fears. This case demonstrates that introducing clozapine can be justified even in chronic patients where improvement may seem unlikely. New monitoring guidelines, which simplify hematological follow-up—requiring neutrophil count checks every three months after the first year of treatment and only once per year after two years of stability—are expected to reduce clinicians’ reluctance and enable broader use of this remarkably effective medication.

**Disclosure of Interest:**

None Declared

